# Mindfulness-based controlled breathing and the effect on performing a transnasal endoscopy: a quasi-experimental study

**DOI:** 10.3389/fmed.2026.1748747

**Published:** 2026-03-30

**Authors:** Lei Sun, Xiaona Sun, Zhishen Zhang, Xinyi Zhang

**Affiliations:** Qingdao Central Hospital, University of Rehabilitation Sciences, Qingdao, China

**Keywords:** discomfort, mindful respiration techniques, nasal endoscopic examination, patient experience, pre-post intervention study

## Abstract

**Purpose:**

Transnasal endoscopy is the traditional modality of choice to evaluate the upper gastrointestinal tract, and patient anxiety and the unpleasantness of medical procedures remain a major problem in clinical practice. In this study, we examined whether mindful breathing can improve patients’ wellbeing before nasal endoscopy.

**Methodology:**

This prospective, non-randomized controlled trial was conducted between January and March 2024 in the international outpatient endoscopy unit of Qingdao Central Hospital, University of Health and Rehabilitation Sciences (Qingdao Central Hospital). A total of 249 adult patients scheduled for transnasal esophagogastroduodenoscopy (EGD) were enrolled. Participants were allocated to either the intervention group or the control group based on their order of arrival and personal preference. The intervention group comprised 124 patients who received a structured mindful breathing protocol, while the control group consisted of 125 patients undergoing standard care without additional breathing guidance. The intervention included pre-procedural instruction, directed breathing before and during the procedure with ongoing coaching. Primary outcome was pain intensity, measured using the Numeric Rating Scale (NRS, 0–10) at three time points: pre-procedure, intra-procedure, and post-procedure. Secondary outcomes included the severity of nausea and vomiting, categorized into four grades, and subjective wellbeing, assessed using a four-point Likert scale. All assessments were recorded by independent observers blinded to group allocation.

**Results:**

The statistical results showed significant difference in both groups. In the intervention group, after practicing mindful breathing, participants’ mean pain level was significantly lower than before they practiced it (2.00 ± 1.05 versus 2.67 ± 1.46, with statistical significance at *P* < 0.001), with a greater percentage experiencing only minor discomfort (95.97% versus 84.80%, *P* = 0.005). The intervention group also showed decreased nausea and vomiting symptoms (1.13 ± 0.53 versus 1.34 ± 0.72, *P* = 0.01), with a higher number of cases rated as mild (93.55% versus 76.80%, *P* < 0.001). Regarding patient comfort, the mindfulness group scored consistently better both prior to (3.01 ± 0.49 vs. 2.77 ± 0.65, *P* < 0.001), (3.03 ± 0.48 vs. 2.69 ± 0.59, *P* < 0.001), but not following examination (*P* = 0.076). The correlations persisted with adjustment of potential confounders in multiple regression analysis.

**Conclusion:**

Mindfulness breathing correlates to a significant decrease of procedure-related pain, particularly in the reduction of pain and GI discomfort with transnasal endoscopy. Such a finding is evidenced-cost-effective technique is a promising method to improve patients’ acceptance, and it may need to be adopted into routine practice by the endoscopic nurses.

## 1 Introduction

Upper gastrointestinal diseases such as Esophagitis, Gastritis, and Duodenitis, are highly prevalent globally (1). In 2019, more than 443 million cases of GI diseases were reported in the world, which has increased by nearly 74% over the past 20 years (since 1990) (2, 3). Upper gastrointestinal endoscopy is an important tool in both diagnosis and management of such disorders (4, 5). Nasal endoscopy is currently used more frequently because it can be performed with fewer errors than other methods of endoscopy and allows for a higher degree of accurate diagnosis (6).

However, the process of gastroscopy often causes a physiological stress response by stimulating the sympathetic nervous system (7) and this can result in transient physiological changes, e.g., increased heart rate, elevated BP and variations of respiratory pattern (8, 9) which are typically brief and transient, suggesting a role of the SNS rather than chronic disease pathology. Sometimes people may have vasovagal episodes (sudden decreases in heart rate and blood pressure), which may result in dizziness or fainting (10). In addition to the above-mentioned autonomic reactions, several patients also report a high degree of pain, including pain, nausea and vomiting during the examination. Such complications may increase anxiety levels, decrease cooperation and satisfaction of the patients with their health care and reluctance for undergoing the same investigations in future if required. Patient’s cooperation is an essential factor that requires a lot from the nurse in order to make the process of examination comfortable for him/her.

Mindfulness is a type of relaxation that comes out of the meditative tradition (11, 12) involving attention to the current moment with acceptance and non-judgmental attitude. Mindfulness interventions could be helpful for reducing distress among patients, decreasing anxiety, and improving quality of life. In the last few years, mindfulness meditation is increasingly being used and studied in China (13, 14). However, there are no studies of its use before gastroscopy. The aim of the present review is to determine if DBs can reduce pain, nausea/vomiting occurrence and patient’s comfort level - in three time points (before treatment, intra-treatment, and after transnasal gastroscopy). The main aim was to determine if this low-cost, non-pharmacological measure would improve patients’ perception and should be considered for inclusion in routine endoscopy nursing practice.

## 2 Materials and methods

### 2.1 Study design and patients

This study employed a non-randomized, non-equivalent control group design. Participants were assigned to either the mindfulness breathing intervention group or the control group according to two sequential criteria: (1) chronological order of arrival for scheduled transnasal EGD procedures, and (2) explicit voluntary choice following an oral explanation of the study conditions. Patients arriving earlier in the enrollment period were more likely to be offered participation, and those who expressed willingness to engage in the breathing protocol were allocated to the intervention group; all others received standard care and were assigned to the control group. To reduce potential selection bias, the eligibility screening was consistently applied to all consecutive patients meeting predefined inclusion criteria during the study period (January–March 2024); no patient was excluded based on demographic characteristics, comorbidities, or anticipated procedural difficulty unless clinically contraindicated. Due to nature of this intervention blinding participants and health care professionals to group allocation was not possible however, the assessors (nurses) who evaluated outcomes were blinded to allocation, and not involved in treatment provision.

The inclusion criteria were as follows: (1) age ≥ 18 years old; (2) good cardiac, respiratory conditions and stable physiologic indicators; (3) Patients who had been planned to undergo nasal endoscopy of the stomach. Excluded individuals were those with (1) cognitive dysfunction; (2) severe cardiovascular or pulmonary illness; (3) allergy to drugs; (4) active peptic ulceration; and/or (5) prior history of nasal surgery. In contrast to those suffering from chronic rhinologic disorders that affect ventilation of the nasal cavity (e.g., allergic rhinitis, chronic rhinosinusitis, or nose polyps) were not automatically excluded but we did not try to include them disproportionately in our study population; no one who signed up for this study had an ongoing pulmonary infection or severe pulmonary disease at time of testing. Institutional Ethics Committee of Qingdao Central Hospital approved the study (KY202400801) with the signature of informed consent from each participant in the studies.

We base our analyses on a study design which follows the reporting guidelines of STROBE (Strengthening the Reporting of Observational Studies in Epidemiology) for observational, quasi-experimental studies.

### 2.2 Study protocol

The upper GI endoscopy was performed using a digital gastroscope (FUJINON EG530-NW) with experienced physicians from the nasal route, and in line with standard national guidelines. In the control group patients were treated as per usual care, with direct nasal endoscopy evaluation and no other treatment.

The anxiety-reducing breath (ARB) was developed in collaboration between a psychologist and an endoscopy nurse with the following steps:

#### 2.2.1 Preparatory phase (initiated roughly half an hour prior to the examination)

Setting: Quiet setting for prep before procedure.

Timing: 1 session of 10–12 min.

Procedure: All subjects were given verbal instructions via an audiocassette tape, read by a nationally certified and licensed psychologist. Subjects were instructed to (1) sit comfortably and close their eyes; (2) focus on their breath; (3) inhale for 4 s via the nose; and (4) exhale slowly, over 6 s, through pursed lips; (5) return to breathing with ease every time the mind had drifted away from it.

Teaching: The participants were informed regarding the purpose of this method as well as its benefits, and asked to simulate before surgery.

#### 2.2.2 Procedural support

At the time of endoscopy, all participants were asked to continue with their trained breath-hold until insertion and manipulation of the endoscope.

Verbal feedback: The participants were given constant verbal guidance (“Breathe in… Now breathe out…” etc.) for correct performance of the breathing exercise by a medical practitioner throughout the experiment.

#### 2.2.3 Post-procedure protocol

Educational resources were distributed, and participants were asked to complete evaluation forms regarding their experience.

No participant in either treatment arm received analgesic, antiemetic, anesthetic, or other drugs during this process and all participants continued to take their usual chronic medications, e.g., medications such as anti-hypertensives, insulin, or chronic gastritis, which cannot be discontinued prior to testing).

## 3 Results

Information regarding participants’ personal characteristics, medical background, and survey answers was gathered through self-reported questionnaires.

### 3.1 Primary outcome

Pain intensity was measured by means of the Numeric Rating Scale (NRS) with a score ranging from 0 to 10, where 0 stands for no pain at all while 10 refers to the worst possible pain imaginable (15). This score was categorized in mild (1–3), mild (4–6), and strong (7–10).

### 3.2 Additional findings

Nausea and vomiting episodes were divided into four categories according to occurrence frequency: Level I (0–1 instances), Level II (2–3 instances), Level III (4–5 instances), and Level IV (6 or more instances) (16, 17).

Comfort level of patients: The investigators developed a personalized four-point Likert type survey questionnaires according to real-life situations, was used for measuring comfortability where respondents were asked “How comfortable are you right now?” with following options: 1 = Very uncomfortable, 2 = Moderately uncomfortable, 3 = Comfortable, 4 = Very comfortable. Ratings were obtained before surgery, during surgery, and after surgery. A limitation of the present research is that this measure has not been subjected to any psychometric testing.

### 3.3 Data processing

All statistical analysis was performed using the IBM SPSS Statistics program (Version 24.0, Armonk, NY). Based on preliminary data (effect size Cohen the = 0.5, α = 0.05, power = 0.8), a minimum of 114 participants per group was required. IBM SPSS Statistics v24.0 (Armonk, NY, United States) with two-tailed *P* < 0.05 considered statistically significant. Continuous variables (age, NRS scores) were tested for normality using the Shapiro-Wilk test. Independent *t*-tests for Normally distributed data; non-normally distributed data using Mann-Whitney U tests; Categorical variables (sex, disease history) using Chi-square or Fisher-Whitney Uested for normality using the Shapiro-Wilk test. Independent refers to analyzed using analysis of covariance (ANCOVA), adjusting for age, sex, and baseline anxiety scores. Comfort scores over time: Repeated-measures ANOVA with Bonferroni correction for multiple comparisons. Multiple imputation (five imputations) for ≤10% missing values; complete-case analysis otherwise.

In addition to these bivariate analyses we ran a set of multivariable regression models to test if there is an independent impact of the mindfulness breathing treatment condition on the outcomes related to pain and comfort scores. Regarding nausea and vomiting severity ordinal logit regressions. Several covariates were controlled for in the regression analyses, including participants’ age, sex (biological), education, and prior disease status. We conducted additional subgroup analysis by gender, including the stratified *t*-test and interactions with *P < 0.05, **P < 0.01, and ***P < 0.001 for two tailed statistics.

### 3.4 Findings

There were 249 patients in the study cohort (mean age: 51.16 ± 10.73; 152 male) without any difference among the groups for the initial clinical variables (age, demographics, gender ratio, education level, etc.,] or medical condition (such as diagnosed with chronic gastritis and reflux esophagitis) in the experimental group and control group [*P* all >0.05 (Table 1) to balance baseline features among treatment groups].

**TABLE 1:** Basic characteristics.

Variables	Control group (*n* = 125)	Mindfulness breathing group (*n* = 124)	*P*
Age (years)	51.14 ± 11.12	51.18 ± 10.36	0.98
Gender			0.66
Male	69 (55.20%)	83 (66.94%)	
Female	56 (44.8%)	41 (33.06%)	
Education levels			>0.05
Below junior high school	23 (18.40%)	29 (23.39%)	
Senior high school	41 (32.80%)	48 (38.71%)	
Junior college or above	61 (48.80%)	47 (37.91%)	
Disease history			>0.05
Chronic gastritis	89 (71.20%)	78 (62.90%)	
Reflux esophagitis	21 (16.80%)	35 (28.23%)	
Others	15 (12.00%)	11 (8.87%)	

### 3.5 Key and supplementary results

The analysis showed significant effect of interventional groups, that is subjects who received mindfulness breathing interventions had significantly lower mean score for pain than those did not receive any treatment (2.00 ± 1.05 vs. 2.67 ± 1.46, *P* < 0.001). Moreover, more mindfulness participants indicated they had felt slightly painful (95.97% versus 84.80%, *P* = 0.005), as detailed in Table 2.

**TABLE 2:** Pain scores.

Pain	Control group (*n* = 125)	Mindfulness breathing group (*n* = 124)	*P*
Scores	2.67 ± 1.46	2.00 ± 1.05	<0.001
Mild pain	106 (84.80%)	119 (95.97%)	0.005
Moderate pain	15 (12.00%)	3 (2.42%)	<0.001
Severe pain	4 (3.20%)	2 (1.61%)	<0.01

Similar results were found for the symptoms of nausea and vomiting. The mindfulness group had a significantly greater improvement, with lower average severity score (1.13 ± 0.53 vs. 1.34 ± 0.72, *P* = 0.01) and proportion of mild disease (93.55% vs. 76.80%, *P* < 0.001), see Table 3.

**TABLE 3:** Nausea and vomiting scores.

Nausea and vomiting	Control group (*n* = 125)	Mindfulness breathing group (*n* = 124)	*P*
Scores	1.34 ± 0.72	1.13 ± 0.53	0.01
Level I	96 (76.80%)	116 (93.55%)	<0.001
Level II	21 (16.80%)	2 (1.61%)	<0.001
Level III	3 (2.40%)	4 (3.23%)	<0.05
Level IV	5 (4.00%)	2 (1.61%)	<0.01

Self-reported comfort was significantly higher in the intervention arm both before the examination (3.01 ± 0.49 vs. 2.77 ± 0.65, *P* < 0.001) and under the examination (3.03 ± 0.48 vs. 2.69 ± 0.59, *P* < 0.001). However, no significant difference was observed between the two groups after the examination (3.63 ± 0.49 vs. 3.51 ± 0.55, *P* = 0.076), which is tabulated in Table 4.

**TABLE 4:** Comfort levels.

Comfort scores	Control group (*n* = 125)	Mindfulness breathing group (*n* = 124)	*P*
Before the examination	2.77 ± 0.65	3.01 ± 0.49	<0.001
Under examination	2.69 ± 0.59	3.03 ± 0.48	<0.001
After the examination	3.51 ± 0.55	3.63 ± 0.49	0.076

### 3.6 Multivariable analysis

Controlling for demographics (age, gender, education level, and comorbidities) we found that the multivariate regression analysis revealed that there were still significant associations of mindfulness breathing with better outcomes, including the intervention were associated with less severe pain (β = −0.63, 95% CI: −0.98 to −0.28, *P* < 0.001), fewer episodes of nausea/vomiting (OR = 0.41, 95% CI: 0.23–0.72, *P* = 0.002), higher procedural comfort (β = 0.32, 95% CI: 0.17–0.47, *P* < 0.001).

### 3.7 Subgroup analysis by gender

**FIGURE 1:**
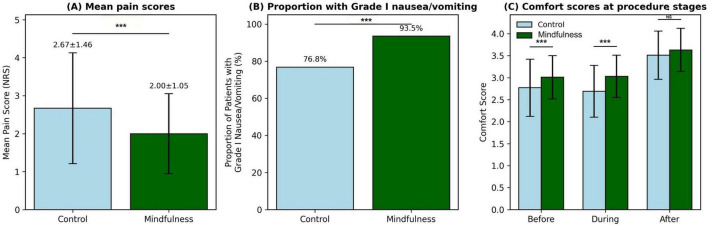
Comparison of key outcomes between the mindfulness breathing group and control group. **(A)** Mean pain scores measured by the Numeric Rating Scale (NRS). **(B)** Proportion of patients reporting Grade I (0–1 episodes) nausea/vomiting. **(C)** Comfort scores assessed before, during, and immediately after the procedure. Error bars represent standard deviations. *P < 0.001; **P < 0.01; ***P < 0.001; ns, non-significant.

Further gender-based analysis showed that , whether male (2.03 ± 1.08 vs. 2.71 ± 1.49, *P* = 0.002) or female (1.95 ± 1.01 vs. 2.61 ± 1.42, *P* = 0.009), the pain scores in the mindfulness breathing group were significantly lower than those in the control group. As no two-way effect-of-group by-sex interactions was observed (*P* = 0.67), the efficacy of treatment did not differ between male and female patients (Table 5). We graphically represent these results in Figure 1.

**TABLE 5:** Subgroup analysis of pain scores by gender.

Subgroup	Control group (*n* = 125)	Mindfulness breathing group (*n* = 124)	*P*	P for interaction
Male	2.71 ± 1.49 (*n* = 69)	2.03 ± 1.08 (*n* = 83)	0.002	0.67
Female	2.61 ± 1.42 (*n* = 56)	1.95 ± 1.01 (*n* = 41)	0.009	

## 4 Discussion

Our findings suggest that conscious breathing has significant positive association to pain relief, reducing the number of patients’ feelings of nausea/vomiting; improving the quality of life of patients during transnasal gastroscopy examination. The results indicate that it is worth including mindful techniques in the routine nursing care of patients undergoing endoscopy procedures.

In our study, there was a strong relationship with reduced perception of pain in relation to the application of concentration breathing: nausea, vomiting. In recent years, breathing techniques have been used in the clinic to alleviate these symptoms as an adjunct therapy (18, 19). Research suggests that mindfulness meditation and relaxation practices induce brain activity associated with positive emotions, thus reducing physiological responses toward pain stimulus (20, 21). Furthermore, directed deep breathing could possibly counteract the somatic reactions to aversive events by influencing parasympathetic/vagal activity balance (22), which in turn may help improve patient tolerance and comfort during endoscopy, which may result in a steadier state of the body’s functions (23).

Moreover, this study also showed a relationship between mindful breathing and higher perceived procedural comfort in patients. Mindfulness could counteract the negative impact of stress on cognition, support brain plasticity, and induce physical relaxation (24). The two-way communication of the patient with their doctor while performing respiration could even have a positive effect on mood and increase trust into health care professionals (25). If they are accompanied by real-time encouragement and motivational comments, they may experience a sense of increased care and individualization that could improve their view of the health services provided to them.

It is interesting that in this survey, both control and experimental group gave similar score for their satisfaction after interview. It may due to feeling of relaxation from all respondents at the end of the interview process, that may be masking any relative benefit of the intervention. On the other hand, this small effect size could represent an inability to measure it, because both groups had comparable post-procedure comfort scores of 3.51 ± 0.55 and 3.63 ± 0.49, respectively.

The baseline patient characteristics were similar in all arms, but because of the observational design of our trial there is a possibility that confounding by unknown factors could have occurred. Factors not evaluated such as mental state (anxiety level, sensitivity to pain), pre-exposure to endoscopy, and variability between individuals may also have influenced the results. These potential confounding factors should be addressed in future studies using methods such as multiple regression analyses and/or marginal structural models (MSMs) or propensity scores, respectively.

### 4.1 Study constraints

This study has certain limitations that need consideration: first is the absence of randomized design in this study may lead to bias sampling for respondents, although all the study arms had comparable baseline features. Second, critical endpoints such as pain scores, incidence of nausea and vomiting, and comfort scores were all self-reported by subjects who can be subject to different interpretations of the same information. Third, the comfort measure was an original and previously unpublished one-item questionnaire developed for the purposes of this study, which suggests that those specific outcomes need to be carefully examined. Fourth, although this study focused on PROs, that excluded objective biological parameters such as cardiac data (heart rate, blood pressure). Future studies should include some type of subjective as well as objective data for a more comprehensive analysis of the effects of treatment. Last, the research did not systematically report on baseline upper airway disease (e.g., rhinitis, sinusitis), which may impact nasal tolerance of an endoscope passed via the nose, and a topic of interest for further research.

It would also be helpful for the validity of this study if we can classify subjects according to nasal/respiratory diseases and see whether there is any difference among them with respect to treatments. The participants in the mindfulness breathing condition received additional clinical time and one-on-one assistance with their clinicians (psychiatrists, psychologists) and nurses; this could affect study results on its own apart from the breathing practice. Further studies need to include a control group with comparable one-on-one contact, but no meditation in order to isolate and better quantify the effect of the treatment. A further limitation is the absence of information on current medications taken by patients, which might influence the outcome (symptoms and/or physiological responses). Future research needs to include a more detailed report of drug intake in order to avoid possible confounding effects.

## 5 Conclusion

Overall, we find an association between breath awareness and pain reduction: reduce the incidence of vomiting and nausea and improve quality of life in patients during transnasal endoscopy. The findings suggest that we should consider implementing this simple yet cost-effective strategy into routine endoscopic nursing practice to improve the experience of our patients.

## Data Availability

The original contributions presented in this study are included in this article/supplementary material, further inquiries can be directed to the corresponding author.
